# The Effect of Kanamycin and Tetracycline on Growth and Photosynthetic Activity of Two Chlorophyte Algae

**DOI:** 10.1155/2016/5656304

**Published:** 2016-09-25

**Authors:** Khawaja Muhammad Imran Bashir, Man-Gi Cho

**Affiliations:** Department of Biotechnology, Division of Energy and Bioengineering, Dongseo University, Busan, Republic of Korea

## Abstract

Antibiotics are routinely used in microalgae culture screening, stock culture maintenance, and genetic transformation. By studying the effect of antibiotics on microalgae growth, we can estimate the least value to inhibit growth of undesired pathogens in algal culture. We studied the effect of kanamycin and tetracycline on the growth and photosynthetic activity of two chlorophyte microalgae,* Dictyosphaerium pulchellum* and* Micractinium pusillum.* We measured CFU mL^−1^ on agar plates, optical density, fluorescence yields, and photosynthetic inhibition. Our results showed a significant effect of* kan* and* tet* on the tested microalgae species except* tet,* which showed a minor effect on* M. pusillum.* Both antibiotics are believed to interact with the protein synthesis machinery; hence, the inhibitory effect of the tested antibiotics was further confirmed by isolation and quantification of the whole cell protein. A significant reduction in protein quantity was observed at concentrations more than 5 mg L^−1^, except* M. pusillum*, which showed only a slight reduction in protein quantity even at the maximum tested concentration of* tet* (30 mg L^−1^). This study can further aid in aquaculture industry, for the maintenance of the microalgae stock cultures and it can also help the microalgae genetic engineers in the construction of molecular markers.

## 1. Introduction

Microalgae are gaining importance in medical, pharmaceutical, and food industry. With the increasing applications of microalgae, it is mandatory to investigate growth conditions and potential growth inhibitors. Herbicides, antibiotics, and heavy metals are toxic to microalgae even at low concentrations [1–6]. Studying the survival and adoption of microalgae in the contaminated environment is not an insignificant question and to a certain extent, the microalgae could survive in contaminated environments [7–10].

In the past decade antibiotics use and resistance have been the focus of the world leading organizations, including the Center of Disease Control (CDC) and the World Health Organization (WHO). Alexander Fleming and Howard Walter Florey warned the world first time about the antibiotic resistance while receiving 1945 Nobel Prize for the discovery of penicillin [11]. Antibiotic resistance has been a productive research topic for scientists in the medical field [12]. Anthropogenic activities including use of antibiotics in agriculture, aquaculture, and waste disposal have been linked with the antibiotic resistance [13–15].

Aminoglycosides are the commonly used broad-spectrum antibiotics, that is, streptomycin, kanamycin, and amikacin. Aminoglycosides are characterized as multifunctional hydrophilic carbohydrates with several amino and hydroxyl activities having higher affinities to the prokaryotic rRNA [16, 17]. Suzuki et al. studied the effect of kanamycin on bacterial protein inhibition [18]. Kestell et al. reported the effect of kanamycin and streptomycin on the macromolecular composition of* Escherichia coli* strains [19]. The inhibitory effect of streptomycin had been reported to microalgae species at a concentration of 0.5 to 150 mg L^−1^ [20–22]. Galloway reported a halotolerant algae* Amphora coffeaeformis* resistance to streptomycin [23]. Kvíderová and Henley reported the effect of ampicillin and streptomycin on the growth and photosynthetic activity of halotolerant chlorophyte algae species [24]. However, a limited or no literature is available on the structural studies of aminoglycosides interaction with RNA sequences.

Kanamycin is a broad-spectrum aminoglycoside antibiotic, isolated from bacterium* Streptomyces kanamyceticus* [25]. It is considered an important medication needed in a basic health system and it has been listed in the WHO's list of Essential Medicines [26]. Kanamycin interacts with the 30S ribosomal subunit resulting in a significant amount of mistranslation and prevents translocation during protein synthesis [27, 28], whereas tetracyclines bind to the 16S part of the 30S ribosomal subunit and prevent amino-acyl tRNA to attach at A-site of mRNA-ribosome complex, ultimately inhibiting protein synthesis as well as cell growth [29–31].

Kanamycin resistance (*Kan* ^*R*^) is mainly due to the cytoplasmic aminoglycoside phosphotransferase that inactivates kanamycin by covalent phosphorylation. On the other hand, tetracyclines are a group of broad-spectrum antibiotics, but their general application has been shortened because of the inception of antibiotic resistance [32–34]. Cells can become resistant to tetracyclines by one of the three mechanisms: enzymatic inactivation of tetracycline, efflux, and ribosomal protection [35].

Antibiotics tolerance of prokaryotic microorganisms has been described by leading scientists, but there are just a few reports available on the antibiotic tolerance study of eukaryotic microalgae [20, 22, 23, 36]. No doubt, antibiotics are normally considered effective against prokaryotic microorganisms, but they are extensively used in microalgae culture screening [37, 38], in aquaculture, and for screening of genetic transformants [39]; hence, there is a need to check the effects of the antibiotics against eukaryotic microalgae.

This work was planned to determine the activity of two important antibiotics, kanamycin sulfate and tetracycline hydrochloride, against the freshwater eukaryotic microalgae species,* Dictyosphaerium pulchellum* and* Micractinium pusillum*. Colony forming units, optical density, fluorescence yields, and photosynthetic inhibitions were measured. The antibiotics used in this study are believed to interact with the protein synthesis machinery; hence, the whole cell protein was also extracted and quantified.

## 2. Material and Methods

### 2.1. Microalgae Cultivation and Treatment

The eukaryotic freshwater microalgae species,* Dictyosphaerium pulchellum* and* Micractinium pusillum,* used in this study were obtained from the Korea Marine Microalgae Culture Center (KMMCC), Busan, South Korea. Stock cultures were stored on the modified AF6 agar slants [40]. The cultures were streak plated and purified by subculturing by at least 5-6 times before use. Both microalgae species were cultivated in 250 mL flasks with 150 mL, modified AF6 medium while incubating at 25 ± 2°C, 50 ± 2 *μ*mol photons m^−2^ s^−1^ and 50% humidity. Antibiotics, kanamycin sulfate (Amresco), and tetracycline hydrochloride (Bio101) with different concentrations ranging from 0 to 30 mg L^−1^ were used. Growth rates were calculated by measuring the absorbance at 750 nm (OD_750_) on every alternating day [41]. Additionally, all the experiments were repeated three times.

### 2.2. Screening Tests

The spread plate method according to Markham and Hagmeier [42], with slight modifications, was used to obtain colonies of the tested microalgae on agar plates. 200 *μ*L of the cultured microalgae with approximately adjusted initial cell density (1 × 10^4^ cells mL^−1^) was spread plated on AF6-agar plates supplemented with different concentrations of* kan* and* tet* ranging from 0 to 30 mg L^−1^. Plates were incubated under constant light intensities and the growth was observed for three weeks.

### 2.3. Modulated Fluorescence and Photosynthetic Inhibition Measurement

Fluorescence yields of algae samples treated with different concentrations of* kan* and* tet* were measured by toxy-PAM dual channel yield analyzer (Heinz Walz GmbH, Effeltrich, Germany). The toxicity test is based on extremely sensitive measurement of the effective quantum yield (*Y*), of photosystem II (PSII), via assessment of chlorophyll fluorescence yield by following the saturation pulse method [43, 44]. Fluorescence of the dark adopted algal samples (*F*_0_) is measured by using modulated light of low intensity to avoid the reduction of the PSII primary electron acceptor (*Q*_*A*_) [43]. In order to induce an equilibrium state for the photosynthetic electron transport, prior to measurement of fluorescence, algal cells were adapted to darkness for 20 min.

In the toxy-PAM blue light is used for excitation and fluorescence is assessed at a wavelength above 650 nm. The (*F*_0_) fluorescence level corresponds to the fluorescence measured shortly before the application of a saturation pulse. Maximum fluorescence level (*F*_*m*_) corresponds to the maximal fluorescence measured during a saturation pulse. The effective PSII overall quantum yield of the photochemical energy conversion was calculated by the formula given by Genty et al. [44].

(1) $$\begin{array}{c} Y=\text{Yield}=\frac{\left(F_{m}-F_{0}\right)}{F_{m}}=\frac{F_{v}}{F_{m}}. \end{array}$$

Relative photosynthetic inhibition of the investigated samples with respect to the reference sample was calculated by the following formula:

(2) $$\begin{array}{c} \text{Relative Photosynthetic Inhibition}\%=\frac{100\left(Y_{2}-Y_{1}\right)}{Y_{2}}. \end{array}$$

### 2.4. Protein Isolation and Quantification

The tested antibiotics are believed to interfere with the protein synthesis machinery; hence, at the end of the experiment, the whole cell protein was isolated by total protein extraction kit (Invent Biotechnologies). The extracted protein was quantified by BCA protein quantification assay kit (Pierce Biotechnology), while bovine serum albumin (BSA) was used as a standard. The extracted protein was electrophoresed on SDS-PAGE with 30% acrylamide : bisacrylamide solution and dyed for 1 h with coomassie brilliant blue G-250 (sigma). The gels were destained overnight with destaining solution and documented.

## 3. Results

**Figure 1:**
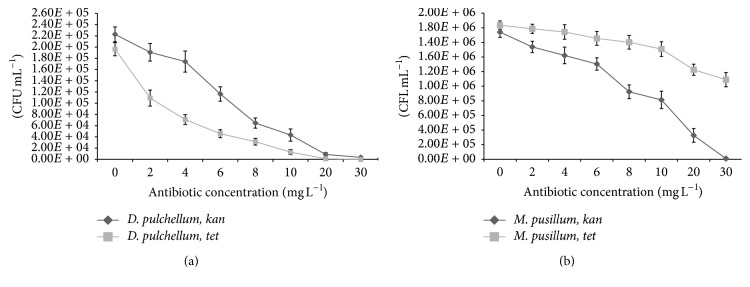
(a) Colony forming units of* D. pulchellum* on* kan* and* tet* agar plates. (b) Colony forming units of* M. pusillum* on* kan* and* tet* agar plates. *X*-axis represents concentrations of antibiotics and colony forming units (CFU) per mL^−1^ are shown along the *y*-axis. Values are means ± SE, *n* = 3.

**Figure 2:**
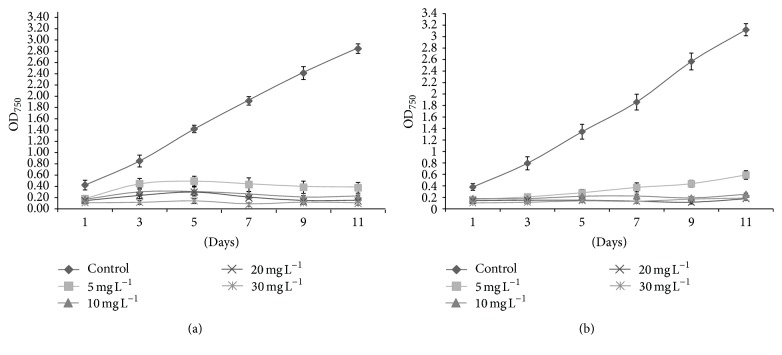
(a) Growth of* D. pulchellum* in AF6 medium supplemented with* kan*. (b) Growth of* D. pulchellum* in AF6 medium supplemented with* tet*. *X*-axis represents days and absorbance values at 750 nm (OD_750_) are shown along the *y*-axis. Values are means ± SE,* n *= 3.

**Figure 3:**
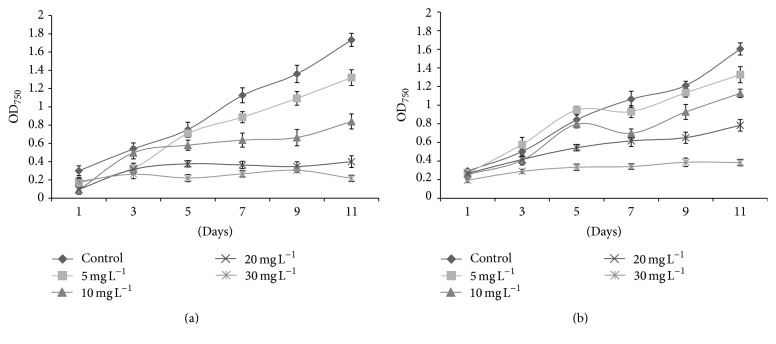
(a) Growth of* M. pusillum* in AF6 medium supplemented with* kan*. (b) Growth of* M. pusillum* in AF6 medium supplemented with* tet*. *X*-axis represents days and absorbance values at 750 nm (OD_750_) are shown along the *y*-axis. Values are means ± SE, *n* = 3.

During this study, antibiotic sensitivity of two freshwater eukaryotic microalgae was assessed. Microalgae species showed significant sensitivities to the tested antibiotics as indicated by their colony forming units, fluorescence yields, and protein concentrations. The CFU mL^−1^ of* D. pulchellum* reduced significantly with the increasing concentrations of* kan* and* tet*. The CFU mL^−1^ of 3.50 × 10^3^ was observed with* kan* at a concentration of 30 mg L^−1^, but at the same concentration of* tet*, no colony was observed (Figure 1(a)). There was a reduction in CFU of* M. pusillum* with increasing concentration of* tet,* but CFU of 1.09 × 10^6^ was observed even at the maximum tested concentration (Figure 1(b)). A substantial decrease in CFU of* M. pusillum* was observed with increasing concentrations of* kan*. Similar results were achieved with growth measurement study at absorbance of 750 nm (OD_750_) (Figures 2(a), 2(b), 3(a), and 3(b)).

**Figure 4:**
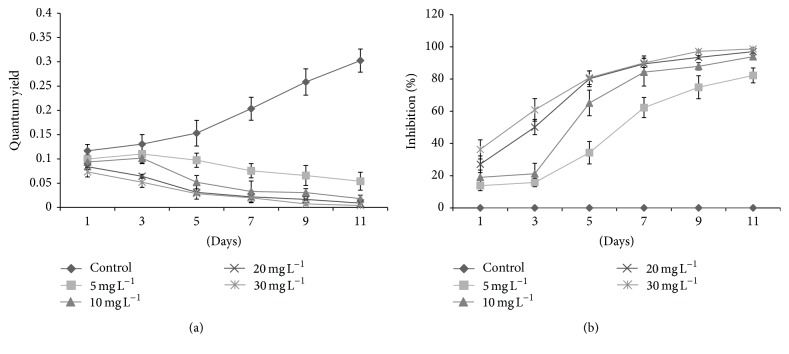
(a) Modulated fluorescence yield of* D. pulchellum* treated with* kan*. *X*-axis represents days and fluorescence yield values are shown along the *y*-axis. (b) Relative photosynthetic inhibition of* D. pulchellum* treated with* kan*. *X*-axis represents days and inhibition percentages are shown along the *y*-axis. Values are means ± SE, *n* = 3.

**Figure 5:**
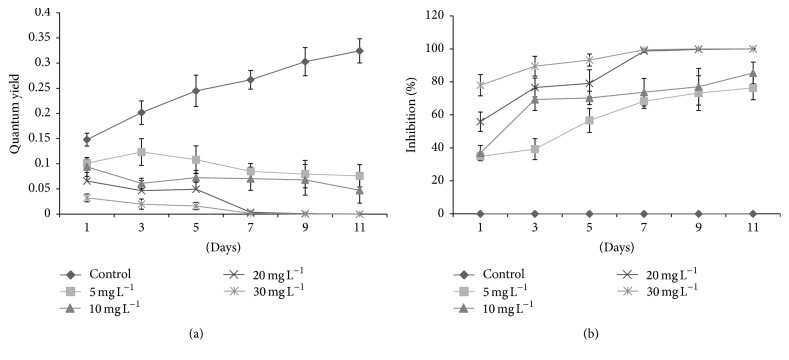
(a) Modulated fluorescence yield of* D. pulchellum* treated with* tet*. *X*-axis represents days and fluorescence yield values are shown along the *y*-axis. (b) Relative photosynthetic inhibition of* D. pulchellum* treated with* tet*. *X*-axis represents days and inhibition percentages are shown along the* y*-axis. Values are means ± SE,* n* = 3.

**Figure 6:**
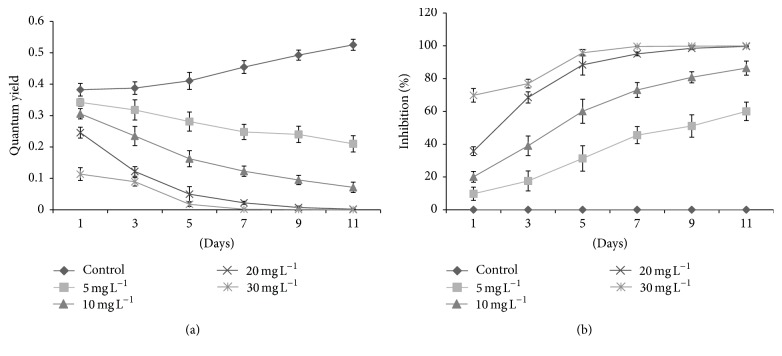
(a) Modulated fluorescence yield of* M. pusillum* treated with* kan*. *X*-axis represents days and fluorescence yield values are shown along the *y*-axis. (b) Relative photosynthetic inhibition of* M. pusillum* treated with* kan*. *X*-axis represents days and inhibition percentages are shown along the* y*-axis. Values are means ± SE,* n* = 3.

**Figure 7:**
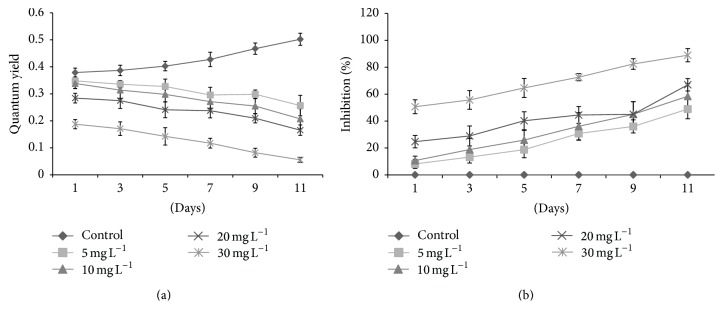
(a) Modulated fluorescence yield of* M. pusillum* treated with* tet*. *X*-axis represents days and fluorescence yield values are shown along the *y*-axis. (b) Relative photosynthetic inhibition of* M. pusillum* treated with* tet*. *X*-axis represents days and inhibition percentages are shown along the *y*-axis. Values are means ± SE,* n* = 3.

The fluorescence yields and photosynthetic inhibition percentages of tested algal species against* kan* and* tet* showed significant variations. Initially,* D. pulchellum* showed a slight increase in fluorescence yield with* kan* and* tet* at concentrations of 5 and 10 mg L^−1^ but after the 3rd day of inoculation, a significant reduction in fluorescence yield was observed with all the tested concentrations as compared to the control (0 mg L^−1^) (Figures 4(a) and 5(a)). This species showed photosynthetic inhibition at all the tested concentrations after the 3rd day of culturing (Figures 4(b) and 5(b)).* M. pusillum* also showed variation in fluorescence yield and photosynthetic inhibition. When treated with* kan*, the concentrations, 20 and 30 mg L^−1^, did not show fluorescence yield even at the 11th day of experiment, but when treated with the same concentration of* tet* it showed a slight variation in fluorescence yields (Figures 6(a) and 7(a)). A variable degree of photosynthetic inhibition percentages was achieved when treated with* kan* and* tet* (Figures 6(b) and 7(b)).

**Figure 8:**
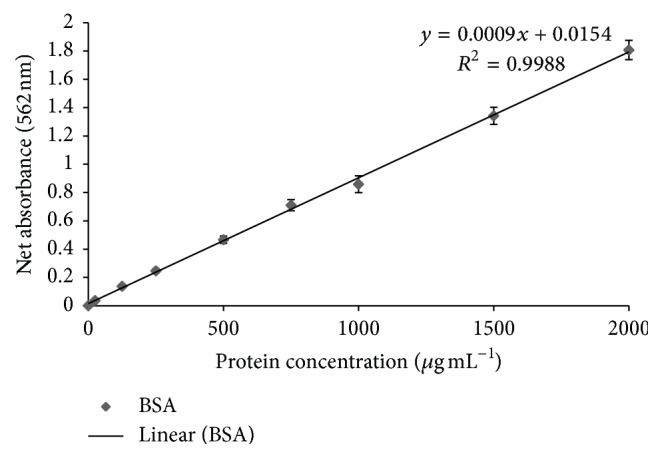
Standard curve for Bovine Serum Albumin (BSA). *X*-axis indicates BSA concentrations and absorbance values at 562 nm are shown along the *y*-axis. Values are means ± SE,* n *= 3.

**Figure 9:**
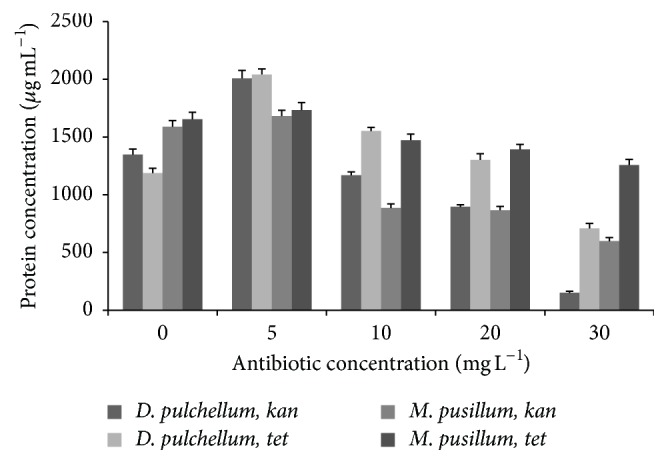
Protein quantities of* D. pulchellum* and* M. pusillum* treated with* kan* and* tet. X*-axis represents concentrations of antibiotics,* kan* and* tet*; *y*-axis represents protein concentrations in *μ*g mL^−1^. Values are means ± SE, *n* = 3.

Whole cell protein from both microalgae species was extracted and quantified by BCA protein quantification assay, while BSA was used as a control. A BSA standard curve was drawn with optical density values at 562 nm versus BSA concentrations (Figure 8). An increase in protein quantity was observed with* kan* and* tet* at a concentration of 5 mg L^−1^, but a significant reduction in protein quantity was observed at the higher concentrations (Figure 9). However,* M. pusillum* showed only a slight reduction in protein quantity even at the maximum tasted concentration of* tet* (30 mg L^−1^).

## 4. Discussion

The antibiotic sensitivity has been reported for different microorganisms, but there is a limited or no literature available on the antibiotic sensitivity characteristics of microalgae. During this study, antibiotic sensitivity characteristics of two freshwater eukaryotic microalgae species* D. pulchellum* and* M. pusillum* were evaluated against the two important protein synthesis inhibiting antibiotics, kanamycin sulfate and tetracycline hydrochloride. The sensitivity of* D. pulchellum* and* M. pusillum* to kanamycin and tetracycline was estimated by colony forming units on agar plates, variation in whole cell protein quantities, modulated fluorescence yields, and relative photosynthetic inhibition percentages. Microalgae species showed significant sensitivities against the tested antibiotics as indicated by their fluorescence kinetics and protein concentrations.* D. pulchellum* showed reduction in growth with both antibiotics; even a clear difference in the extracted protein quantities was observed.* M. pusillum* also showed reduction when tested against* kan* but showed only a slight reduction in growth on* tet* agar plates even at the highest tested concentration (30 mg L^−1^). Interestingly, when absorbance was tested at 750 nm (OD_750_), this species also showed reduction in growth with increasing concentrations of* tet;* however, a minor growth was observed at the maximum tested concentration (30 mg L^−1^). This species may also show inhibition at higher concentrations of* tet*. Both the tested species showed significant reduction in growth at* kan* and* tet* concentrations higher than 10 mg L^−1^. The minimum inhibitory concentration for* D. pulchellum* was recorded as 6 mg L^−1^ with* kan* and 8 mg L^−1^ with* tet,* whereas the minimum inhibitory concentration of* kan* against* M. pusillum* was recorded as 8 mg L^−1^. To further confirm the effect of the tested antibiotics, whole cell protein from both microalgae species was extracted and quantified by BCA protein quantification assay while BSA was used as a standard. The results of isolated proteins were quite interesting; both the tested antibiotics showed increase in protein quantity with* kan* and* tet* at a concentration of 5 mg L^−1^ but a clear reduction in protein quantity was observed at higher concentrations. However,* M. pusillum* showed only a slight reduction in protein quantity even at the maximum tasted concentration of* tet* (30 mg L^−1^). Whether the tested antibiotics at low concentrations accelerated the growth or not cannot be concluded at this stage. Further study and biochemical analyses are required to support the findings. This basic study can further aid the microalgae genetic engineers in construction of molecular markers and in microalgae stock culture maintenance.

## 5. Conclusion

Kanamycin and tetracycline are routinely used for human and animals. The sensitivity of* D. pulchellum* and* M. pusillum* was studied to kanamycin and tetracycline through colony forming units on agar plates, variation in protein concentrations, quantum yields, and photosynthetic inhibition percentages. Both the tested species showed significant reduction in growth at* kan* and* tet* concentrations higher than 10 mg L^−1^ except* M. pusillum* which showed growth even at the maximum tested concentration of tetracycline (30 mg L^−1^). This study can further aid in aquaculture industry, for the maintenance of the microalgae stock cultures, and it can also help the microalgae genetic engineers in the construction of molecular markers.

## Abbreviations

*kan*: Kanamycin
*tet*: Tetracycline
*Y*_1_*⁡*: Fluorescence yield of the investigated sample
*Y*_2_*⁡*: Fluorescence yield of the reference
*Y* = *F*_*v*_/*F*_*m*_: Quantum yield of photosystem II (PSII)
*F*_0_*⁡*: Minimum fluorescence value
*F*_*m*_*⁡*: Maximum fluorescence value
*F*_*v*_*⁡*: Variable fluorescence
SDS-PAGE: Sodium dodecyl sulfate-polyacrylamide gel electrophoresis
BCA: Bicinchoninic acid
BSA: Bovine serum albumin.
